# Desperately Seeking Stable 50-Year-Old Landscapes with Patches and Long, Wide Corridors

**DOI:** 10.1371/journal.pbio.1001253

**Published:** 2012-01-31

**Authors:** Paul Beier, Andrew J. Gregory

**Affiliations:** School of Forestry and Merriam-Powell Center for Environmental Research, Northern Arizona University, Flagstaff, Arizona, United States of America

## Abstract

Conservation corridors are a promising intervention to preserve biodiversity, yet most research has explored corridors in a landscape context different from their intended purpose. This Community Page asks readers to help identify landscapes that will help answer the question: Do corridors work?

## Introduction

Human activities such as urbanization and roads have disrupted movement and gene flow for plants, reptiles, mammals, sedentary birds, and arthropods [1],[2]. Indeed, human-caused habitat fragmentation is a leading threat to biodiversity [3]. As plant and animal populations become smaller and more isolated they become more susceptible to stochastic events and reduced genetic diversity via drift and inbreeding [4]. The primary conservation interventions to counteract habitat fragmentation are conservation corridors and increased reserve size; conservation corridors are also the most frequently cited recommendation to conserve the ability of species and ecosystems to adapt to climate change [5].

Because corridors are such a promising conservation intervention, they are being designed and implemented in many parts of the world. For example, one of us (PB) has helped develop high-resolution plans, each of which is being implemented by government agencies and nongovernmental organizations, to conserve corridors in Bhutan [6], coastal southern California (http://www.scwildlands.org), Arizona (http://www.corridordesign.org/arizona), southeastern California, and northern California. Each corridor (*n  = * 72) is a swath of natural land 500 m to 80 km long identified for conservation so that it can support gene flow and demographic interactions between a pair of natural landscape blocks after “build-out”—i.e., after lands adjacent to the corridor and the natural landscape blocks have been converted to urban, agricultural, or industrial uses that are incompatible with wildlife movement.

Despite the large body of research confirming that corridors promote wildlife movement [7],[8], there is no strong evidence that these 72 corridors will promote gene flow and demographic movement among plants and animals connected by the corridors as intended. Nonetheless, it’s clear that swaths of land much smaller than conservation corridors and embedded in a matrix that is not intensely modified by human activities do support presence and movement of plants and animals [7],[9]. These results suggest that corridors may be a useful conservation intervention. However, evidence that such movements occur often enough to promote genetic connectivity and patch occupancy across longer distances in human-dominated landscapes is generally lacking (but see [10]).

Evidence is lacking primarily because most corridor studies have been conducted in landscapes that are vastly smaller and different than the landscapes conservation corridors are designed for and have measured species response variables weakly related to corridor utility. That’s why we’re issuing a call for help to identify appropriate sites to test the effectiveness of conservation corridors on a global scale.

## The Gap between Corridor Research and Corridor Design

First, almost all corridor research has concerned corridors <150 m long, but proposed and implemented conservation corridors are much longer. Second, in most ecological studies, a corridor is any narrow swath of land connecting two habitat patches where the patches and corridor share a land cover dissimilar from the surrounding matrix, e.g., grassland patches and corridors in a forest matrix [11]. This definition depends only on structural layout of the focal land cover type, regardless of adjacent land uses. In contrast, each conservation corridor is a swath of natural land that is (or eventually may be) embedded in urban, agricultural, or industrial landscapes. Although most conservation corridors are designated for conservation while the matrix is still in a relatively natural state, they are explicitly predicted to be useful for species conservation after build-out.

With respect to response variables, almost all studies of corridor utility document only whether focal species were present in or moved through the corridor [7],[8]. Although presence and movement are necessary for corridor utility, they do not demonstrate that the corridor enhances demographic stability, gene flow, or recolonization—which are ultimately the intended outcome of conservation corridors [12]. Variables that would demonstrate these outcomes include genetic relatedness, reflecting effective connectivity among patches [13], and long-term patch occupancy, reflecting demographic rescue and recolonization [14].

## Help Us Identify Stable, 50-Year-Old Landscapes with Patches and Long, Wide Corridors

We have designed a research project to collect the evidence to determine if conservation corridors work and the conditions (such as width, severity of constrictions, or adjacent land uses) associated with success. Because planned and implemented conservation corridors designed by wildlife planners are too young for genetic and demographic effects to be evaluated, we will study landscapes with “de facto” conservation corridors (i.e., corridors >500 m long, in a human-dominated matrix). A de facto corridor can be thought of as an accidental corridor that exists as a quirk of the way the landscape has been developed. In each landscape (*n* > 50), we will collect DNA samples from focal species in patches connected by corridors, isolated patches, and sampling locations within an intact natural area. A corridor will be deemed successful if genetic distances among connected patches are smaller than genetic distances among isolated patches and similar to genetic distances between sampling sites in intact habitat. Focal species will vary among landscapes and may include any reptile, amphibian, mammal, flightless arthropod, or sedentary bird associated with the patches and corridors, but not the human-dominated matrix. In each landscape, the configuration of patches and corridors must have been stable for at least 20–50 years, so that genetic structure is likely reflective of landscape pattern. (For more information on the study design, see http://www.docorridorswork.org/study-site-criteria/). Unfortunately, we have not yet been able to identify the critical number of landscapes needed to put conservation corridors to the test.

It is the authors’ good fortune to live in the southwestern US, an area with many large, intact natural landscapes, and where fragmentation has occurred too recently for a genetic response to manifest. We need readers and colleagues to help us identify appropriate study systems, where each study system consists of a landscape and focal species. We encourage readers to suggest appropriate sites, or to direct others who might know of appropriate sites, to http://www.docorridorswork.org. Small honoraria are available for informants who provide leads to study systems that meet most of the seven criteria and become part of our study (see Box 1).

**Box 1. Criteria for Potential Conservation Corridor Study Sites**

**Historically continuous habitat.** Prior to human alteration of the landscape, the natural cover types used by the focal species must have been widespread and relatively continuous, as in the “Before” panel of Figure 1A. In other words, we will not study landscapes in which the patches are naturally isolated, such as a group of naturally disconnected marshes.

**Focal species restricted to natural matrix and dependent on corridors for connectivity.** There must be at least one mammal, reptile, amphibian, sedentary bird, or flightless arthropod that is expected to occur in natural patches, but probably cannot disperse through habitat in the matrix. Although bats and flying birds have been shown to travel along linear habitat features, most of them are not suitable focal species because they can maintain demographic and genetic flows without corridors. Focal species will differ among landscapes, and we anticipate that each landscape will have only one or two focal species. Estimated effective population sizes (typically 10% to 20% of census population sizes [15]) in the isolated patches must be low enough for genetic divergence to have occurred during the period of landscape stability since build-out.

**At least one corridor and one reference condition in the landscape.** The landscape must contain at least one corridor and at least one type of reference condition (Figure 1).

**Corridor > 0.5 km long.** Pairs of patches must be separated by distances > 0.5 km, with similar distance between pairs of isolated patches, pairs connected by corridors, and pairs of sampling locales within intact habitat. Each of the previously mentioned 72 conservation corridors is longer than 500 m.

**Corridor > 100 m wide.** The corridor must be >100 m wide (except for short constrictions such as a highway crossing structure). Conservation practitioners never recommend narrower corridors as a conservation intervention [16].

**Landscape stable for >20–50 years.** The configuration of patches, corridors, and matrix must have been stable for at least 20 to 50 years, except for natural disturbances. This duration makes it likely that genetic pattern reflects landscape pattern. Although genetic equilibrium is probably never reached for any population in a human-altered landscape, genetic distances approach equilibrium values quickly after perturbations [17],[18]. More specifically, genetic divergence should be evident after ten generations of the focal species for effective population sizes <50 per patch and after 20 generations for larger effective population sizes [13],[15],[19].

**Matrix dominated by urban, agricultural, or industrial forestry uses.** The matrix should be dominated by urban, industrial, agricultural, industrial forestry, or any other intense land uses. Secondarily, we will consider landscapes in which the matrix is dominated by semi-natural pasture or forests where logging is constrained by ecological goals, but only if there is strong evidence that the human-caused alteration presents a strong barrier to movement of the focal species.

## Other Considerations

A large sample of study systems probably will include some corridors that work, and others that do not. To determine what factors are associated with success or failure of a corridor, we will select study systems that vary with respect to five landscape variables: corridor length, mean corridor width, severity of constrictions in the corridor, type of matrix (e.g., high-density urban, rural residential, intensive agricultural, pasture), and degree of human disturbance (including recreation, artificial night lighting, and vehicle traffic) in the corridor. Because traits of the focal species can also affect observed genetic patterns, our analyses will evaluate corridor effectiveness for multiple species that exhibit a range of additional covariates for traits such as species mobility or edge sensitivity. Strong inferences about five landscape variables and two species variables will require at least 50—preferably 100—replicate study systems.

Perhaps the greatest risk is that all or almost all corridors that meet our criteria will be narrow (e.g., <100 to 500 m wide) or will have severe bottlenecks (e.g., small highway crossing structures) such that very few corridors support gene flow. In other words, Earth may not contain enough successful de facto conservation corridors to identify minimum width or other traits of successful corridors. Nonetheless, simply knowing that the minimum width must be greater than 500 m would conclusively rule out such narrow corridors as a credible conservation strategy. If only a few broad corridors are in the sample, and they all succeeded, the results might not be statistically significant, but they would be biologically relevant and persuasive.

We anticipate many of our study systems will be in Europe, because many European landscapes have been built-out for decades, in contrast to more rapidly changing landscapes in much of the rest of the world. Nonetheless, if we find that corridor utility depends on corridor width, species mobility, or an interaction between corridor width and type of matrix, those results should apply broadly. Our focus on the impact of a handful of high-level, generalizable landscape and species variables ensures that the results will be globally relevant. This focus also gives the proposal a strong foundation in basic science, squarely addressing theoretical issues related to interactions between species life-history strategies and landscapes, the degree to which inferences from small corridors scale up to large corridors and landscapes, and the degree to which presence and movement are reliable indicators of genetic and demographic connectedness.

Conservation scientists need to know what factors are associated with successful wildlife corridors so that they can design and implement effective conservation corridors. Land managers need to know what land uses and management practices are compatible with effective corridors. Ecologists need to know how corridor width, internal characteristics of corridors, characteristics of the matrix in which corridors are embedded, and traits of focal species affect corridor utility. We look forward to engaging and working with many colleagues in a rigorous, global study to address these issues.

**Figure:**
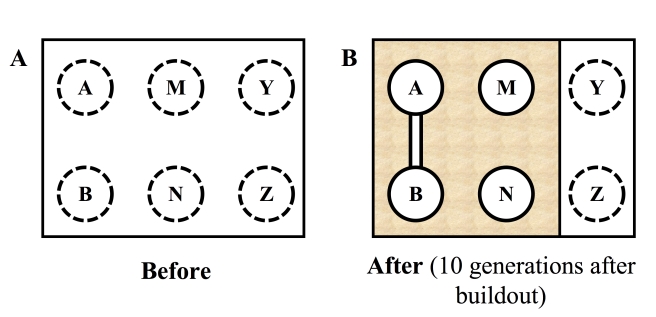
(B) illustrates an ideal study site; white background indicates natural land cover; stippling indicates land converted to urban or agricultural uses incompatible with movement by the focal species. Reference condition 1 consists of habitat patches/blocks separated by approximately the same Euclidean distance as the connected patches, but surrounded by human-altered matrix for at least 10–20 generations of the focal species (B, M–N). The second reference condition is an intact habitat block large enough to allow researchers to obtain genetic samples at sampling sites spaced at approximately the same Euclidean distance from each other as the connected patches as in (B, Y–Z). Ideally a landscape would have both types of reference conditions. In addition, the land cover in the corridors must be similar to that of the patches and large natural landscape blocks.
